# Mixed reality headset in vitreoretinal surgical teaching: a newly defined tele-mentoring model

**DOI:** 10.1186/s40942-026-00829-y

**Published:** 2026-03-14

**Authors:** Rodolfo Mastropasqua, Maria Ludovica Ruggeri, Alberto Quarta, Ruggero Tartaro, Luca Vecchiarino, Lorenza Brescia, Corina Valentina de Sanctis Ciacci, Raffaella Aloia, Matteo Gironi, Ilaria Palladinetti, Matteo Orione, Andrea Russo, Teresio Avitabile, Leonardo Mastropasqua

**Affiliations:** 1 Department of Neuroscience, Imaging and Clinical Sciences, University G. D’Annunzio Chieti-Pescara, Via dei Vestini 31, 66100 Chieti, Italy; 2 Eye Clinic, University Hospital of Chieti-Pescara, Via dei Vestini, 66100 Chieti, Italy; 3 Ophthalmology Clinic, Department of General Surgery and Medical- Surgical Specialties, University of Catania, Catania, Italy; 4 Department of Medicine and Science of Aging, University G. D’Annunzio Chieti-Pescara, Via dei Vestini 31, Chieti, 66100 Italy; 5 Ophthalmology Clinic, Via dei Vestini, 66100 Chieti (CH), Italy

**Keywords:** Artificial intelligence, Mixed reality, Augmented reality, Vitreoretinal surgery, Intraocular surgery

## Abstract

**Purpose:**

To report the benefits of a newly defined tele-mentoring surgical model in vitreoretinal surgery based on a mixed reality headset (MRH) to enhance ophthalmic surgical education.

**Methods:**

A two-step approach was used to define and test the efficacy of the tele-mentoring model designed for surgical teaching. First, the application of the innovative MRH in intraocular surgery was validated for safety and efficacy by an expert surgeon. Secondly, a board of vitreoretinal surgeons designed the tele-mentoring model for surgical teaching, entailing a pre-operative training and the execution of vitreoretinal procedures by a fellow supervised by the mentor through the connection offered by the device. Intraoperative parameters and a satisfaction questionnaire were used to assess surgical safety, efficacy and level of satisfaction for the tele-mentoring teaching method. Outcomes in the model validation were: Surgical safety, defined by (i) the absence of intraoperative complications (ii) the absence of early and (iii) late post-operative complications and surgical efficacy. In addition, in the model application MRH wearability and practicability, the all in one system, impact in the procedure and safety and confidence were investigated. In all cases, time lag and surgery duration were measured.

**Results:**

A total of 40 vitreoretinal surgical procedures were performed by an experienced vitreoretinal surgeon with the aid of the MRH. No intraoperative nor early or late postoperative complications were recorded, and time lag and surgical duration were acceptable. A total of 40 surgical procedures were successfully performed by a fellow with the MRH, with no recorded complications and acceptable time-lag and intraoperative time. The tele-mentoring experience was found to be safe and effective, with overall fellows satisfaction in the surgical teaching method.

**Conclusions:**

Reshaping the mentor-fellow relation in a tele-mentoring dimension boosts the fellow self-confidence and training, preserving patient safety and surgical efficacy and representing a promising tool for surgical education in ophthalmology.

**Supplementary Information:**

The online version contains supplementary material available at 10.1186/s40942-026-00829-y.

## Introduction

The absence of homogeneous training in surgical teaching opens to a wide range of possibilities that influence differences in surgical care. A successful surgery cannot rely on the absence of a strong surgical training able to test and validate surgeon abilities in the most challenging scenarios. Nevertheless, recent studies have outlined current differences in surgical education, shedding light on worldwide inequities in ophthalmic training [1, 2]. 

Currently, Ophthalmology has experienced groundbreaking changes in the clinical practice. The traditional, overall accepted, and successful microscope-based surgery has been implemented with visual and three-dimensional systems that have allowed surgeons to live the surgical practice in a new, immersive modality. Likewise, these systems have shown significant effectiveness in surgical performance, with comparable results in surgical success and patient safety [3]. As a result, this has produced changes in clinical practice, offering ophthalmologists the possibility of operating with new approaches. Besides, it has opened to new updates in surgical teaching. A recent review has outlined the transformative impact of novel technology in ophthalmology education, enhancing learning, diagnostic skills and surgical training [4]. In this setting, Seddon and colleagues have reported the successful results of a 3D-based model for vitreoretinal surgery tele-mentoring [5]. Currently, mixed reality (MR) and augmented reality (AR) systems have significantly raised the possibilities of training for learners with simulators and MR systems spreading out in clinical campuses and universities [6]. Likewise, recent attention has been given to virtual reality (VR) devices in clinical practice, evaluating their role as endpoints in clinical trials [7]. Additionally, Ophthalmic robot systems have shown efficacy in vitreoretinal procedures, holding significant promises in the future of vitreoretinal surgery [8, 9]. 

In our previous work, we analyzed the role of an innovative mixed reality headset (MRH) in intraocular surgery and proposed its use as a tele-mentoring tool in a pilot study, demonstrating overall safety and technique effectiveness and achieving good results in terms of possible mentees’ experience in surgical tele-mentoring [10]. Hence, in this study we aim to deepen the tele-mentoring application of this device in surgical training, by developing and testing a structured tele-mentoring model. The importance of mentoring in surgical growth has overall been acknowledged, reinforcing the constructive interpersonal relationship that is at the base of a successful, productive training [11]. 

A recent study has underscored the efficacy of tele-mentoring and telesurgery as revolutionary applications, despite challenges that still need to be overcome [12]. 

Virtual curricular models, simulators and immersive technologies have shown to maximize training all over the world [13]. In this light, tele-mentoring in surgical training would open a wide range of possibilities creating a new method of surgical teaching, reducing boundaries of space or time and shaping and re-elaborating the mentor- mentee relation. To do so, technique effectiveness and patients safety are requirements that need to be fulfilled.

Therefore, the aim of this study is to (1) define and (2) develop a tele-mentoring surgical program that relies on the use of this innovative device in building the next generation of ophthalmic surgeons.

## Methods

This IRB-approved prospective interventional study included participants undergoing silicone oil removal (ROSO), PPV for vitreous hemorrhage (VH) and epiretinal membrane (ERM) removal surgery between January 2025 and March 2025.

All patients were enrolled among those accessing at the Ophthalmology Clinic of University “Gabriele d’Annunzio”, Chieti-Pescara, Italy. Enrolled subjects were carefully informed about their condition, surgical team, surgical procedure and video collection procedures, which were thoroughly discussed during the consent process. The study adhered to the principles of the Declaration of Helsinki, and all patients provided written informed consent. Exclusion criteria were: Patients under 18 years old, ophthalmological conditions other than the one addressed to surgery.

**Fig. 1:**
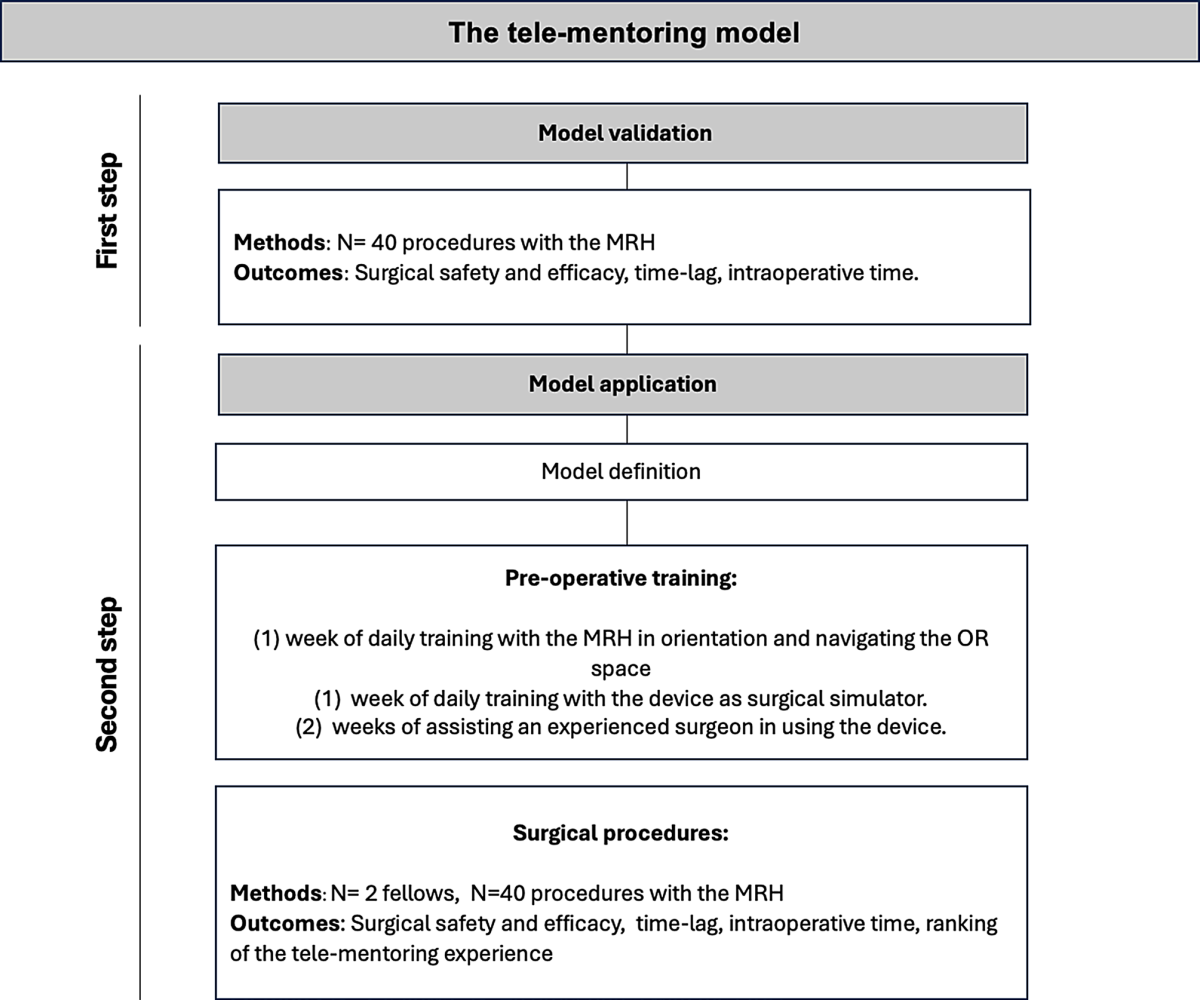
Study flowchart. A two-step approach was used to test safety and efficacy of the tele-mentoring model. MRH: Mixed reality headset

Our two step approach entailed (1) validation of the surgical approach (2) definition and validation of the tele-mentoring model ( Fig. 1). Specifically, our goal was to test: (i) Surgery efficacy and safety (ii) efficacy and practicability of the tele-mentoring model.

### First step- model validation

#### Pre-operative assessment

All patients underwent complete baseline ophthalmological evaluation by undergoing best corrected visual acuity (BCVA), slit lamp biomicroscopy, ophthalmoscopy and intraocular pressure measurement. Additionally, all patients underwent fundus autofluorescence (FAF) and spectral domain optical coherence tomography (OCT) using Spectralis HRA + OCT (Heidelberg Engineering; Heidelberg, Germany). The acquisition protocol entailed a single slice horizontal and vertical scan centered on the fovea, a radial scan and a 49 horizontal raster dense linear B-scans centered on the fovea. Only images with good signal strength (> 25) were included. Additionally, Fundus Photography (Optos, PLC, Dunfermline, Scotland) and biometry was collected in all patients.

In the first step of the model ( model validation), ROSO procedures and ERM removal surgeries were included. Exclusion criteria were any other procedures than the one addressed to the study, age < 18 years old, pregnancy.

#### Surgical procedure

All surgeries were performed by the same, experienced fellowship-trained vitreoretinal surgeon (RM) using the ZEISS ARTEVO^®^ Resight (Carl Zeiss Meditec, Jena, Germany) surgical microscope to perform all the PPVs with the aid of the MRH (Apple Vision Pro, Apple, California).

Before surgery, the surgeon had been training with the device as previously described and had previously performed a number of 9 surgeries with the aid of the MRH [10]. Surgical sterility was uneventfully preserved during all procedures, and the device was worn by the surgeon before scrubbing in, as previously tested [10]. Before surgeries and during procedures, the superimposition of retinography, OCT and optical biometry allowed the surgeon to use additional data during the whole surgery safely.

#### Outcomes

Surgical safety and efficacy were evaluated. Surgical safety was defined by (i) the absence of intraoperative complications (ii) the absence of early post-operative complications, that were defined by a window of 48 h after surgery and (iii) late post-operative complications, that were defined up to one month after surgery.

“Complications” were defined as the presence of intraoperative and/or postoperative events up to 30 days including: iatrogenic breaks, intraocular pressure (IOP) elevation or hypotony, significant/recurrent hemorrhage, endophthalmitis, retinal detachment and any other events that would require the patient need for reoperation.

Efficacy was defined by achieving the desired surgical outcome. Specifically; complete ERM/ILM removal in the case of ERM surgery, evidence of complete ROSO in the case of ROSO, VH resolution in the case of VH surgery.

Additionally, intraoperative parameters were collected: time lag and surgery duration. Surgery duration was measured in minutes and was defined as the operation time from the first incision to the final removal of the speculum. Time lag was measured by considering the difference in frames between the physical move and the screen move when recording the surgical activity on the AVP device and the 3D screen. All frames were checked by two independent examiner and in case of discrepancy a third examiner was consulted.

### Second step- model application

#### Model definition

The tele-mentoring model was developed by a board of three different vitreoretinal surgeons, two of them with previous experience in performing surgery with MRHs. Two vitreoretinal fellows with a minimum of 50 and a maximum of 70 retinal surgeries were selected, according to previous evidence demonstrating approximately 91 vitrectomies required to achieve competency [14]. 

A stepwise approach was defined to allow the fellows to practice and gain confidence with the device. A questionnaire to test the personal experience was developed, inspired by our previous exploratory study [10]. 

#### Pre-operative training

Both fellows underwent a one-month period of training with the MRH before the first surgery to adjust to reported possible symptoms related to the use of the device, such as nausea and difficulty in orientation [15]. As a result, the training period entailed (1) week of daily training with the MRH in orientation and navigating the OR space (1) week of daily training with the device as surgical simulator, (2) weeks of assisting an experienced surgeon in using the device. Vitreous haemorrhage (VH) and ROSO procedures were included. Exclusion criteria were any other procedures than the one addressed to the study, age < 18 years old, pregnancy. Surgical cases were discussed and planned with the help of a different surgeon than the mentor before the surgery.

#### Surgical procedure- the tele-mentoring model

The procedure was completed as follows: On the day of the surgery the mentor, an advanced fellowship-trained vitreoretinal surgeon, was observing and supervising the procedure from a different area of the same hospital, and connected to the OR through the Facetime call. The fellow wore the MRH before scrubbing, to preserve sterility throughout the whole procedure. Before starting the surgery, the Facetime call with the mentor outside the OR was started and continued until the end of the surgery, while pulling the patient multimodal imaging on the shared screen to be discussed and the surgical procedures were then performed (Video 1).

#### Outcomes

The same outcome measures used in the first step were used in the tele-mentoring model. Surgical safety and efficacy were evaluated as previously exposed (ig ROSO in the case of silicone oil in the vitreous chamber and PPV in the case of VH). Additionally, time lag and surgery duration were collected as intraoperative parameters. After the procedure, both fellows were asked about the surgical experience, ranking each item in a scale from 0 to 10. Explored areas were: MRH wearability and practicability, the all in one system, impact in the procedure, safety and confidence, with possibility of specification in each topic. Rankings ≥ 8 were considered to be high, ≥5 but < 8 medium and <5 low.

To explore the mentor – mentee communication and the facetime experience, the resulting mark would derive from internet connection, confidence, sensation of mentor vicinity, feelings transmitted through the MRH interaction both during conversation over Facetime, both resulting from the interaction of the mentor and the fellow with the MRH interface.

## Results

(i) First step: Surgery safety and efficacy

A number of 40 surgical procedures were performed between January 2025 and March 2025 with the aid of the MRH. A total of 20 patients (50%) were female, and 20 (50%) were male. Patients were aged 59 ± 10 years old.

Mean baseline BCVA was 0.3± 0.06 LogMAR in the ROSO group, while 0.52± 0.07 LogMAR in the ERM group.

Surgeries were performed according to the workflow already tested by the surgeon and the OR. Twenty ROSO procedures were performed, and 20 ERM removal surgeries were scheduled and then performed. Among patients undergoing ROSO surgery, 50% were female with a medium age of 60 ± 12. Likewise, 50% of patients undergoing ERM were female, with a medium age of 58 ± 10. Per patient baseline characteristics are reported in Table 1. No intraoperative complications were reported in all cases (100%). Accordingly, no early nor late post-operative complications were reported in enrolled patients. The reintervention/recurrence rate up to one month was 0%. Surgeries showed 100% of efficacy, with successful ROSO and ERM removal in all participants scheduled for the procedure. Mean intraoperative time for ROSO surgery was 30.5 ± 10 min and for epiretinal membrane removal was 51.4 ± 8 min. No difference was found in time lag between the physical move and the screen move. Considering the 3D device time lag of < 50 msec it was considered as acceptable according to previous reports [16, 17].

**Table 1:** Case characteristics and operative time by study phase and procedure

Model validation (*n* = 40)				
ROSO (*n* = 20)	Age *Years*	60 ± 12		
	Sex *Male*	50%		
	Intraoperative time *Minutes*	30.5 ± 10		
ERM removal (*n* = 20)	Age *Years*	58 ± 10		
	Sex *Male*	50%		
	Intraoperative time *Minutes*	51.4 ± 8		
**Model application (** ***n*** ** = 40)**				
ROSO (*n* = 20)	Age *Years*	64 ± 8		
	Sex *Male*	50%		
	Intraoperative time *Minutes*	40 ± 12 min		
VH (*n* = 20)	Age *Years*	62 ± 4		
	Sex *Male*	50%		
	Intraoperative time *Minutes*	35 ± 8 min		

Values are presented as mean and standard deviation. ERM: Epiretinal membrane; ROSO: Removal of silicone oil VH: Vitreous hemorrhage

(ii) Second step: Safety and efficacy in the tele-mentoring model

A total number of 40 surgeries ( 20 ROSO and 20 PPV for VH) were scheduled and then performed. Ten patients in each group were male (50% in each group) with a mean age of 64 ± 8 years in the ROSO group and 62 ± 4 in the VH. Mean baseline BCVA was 0.52± 0.07 LogMAR in the ROSO group, while 1.10± 0.08 LogMAR in the VH group. Surgery was successful and uneventful in all cases (100%) with no intraoperative nor early or late postoperative registered complications nor need for reintervention/recurrence up to one month after surgery. Mean intraoperative time was 40 ± 12 min in the ROSO group, 35 ± 8 in the VH. No difference was found in time lag between the physical move and the screen move. Considering the 3D device time lag of < 50 msec it was considered as acceptable according to previous reports [16, 17]. Both fellows ranked positively the overall experience, with a > 8 rank in all areas (Table 2).

**Table 2:** Questionnaire

Satisfaction Questionnaire Low (< 5) Medium (5–8) High (> 8)	
*The surgical experience*	
The all-in one system	> 8
Advantages in surgical planning and up to date during procedure	> 8
MRH Integration in the OR workflow	> 8
The device maneuverability	> 8
*The Telementoring experience*	
Safety	> 8
Confidence	> 8
Absence of issues or concerns during procedure	> 8
Mentor- Mentee communication	> 8
Facetime experience	> 8
Overall satisfaction in teaching method	> 8

Satisfaction questionnaire and relative results. An overall high level of satisfaction was achieved in all areas

## Discussion

Our analysis shows the efficacy of a newly defined tele-mentoring model for surgical training in Ophthalmology, filling the existent gap in the use of innovative devices in surgical teaching [1]. To do so, we first validated the efficacy and safety of the MRH-integrated surgery, to develop and test the innovative tele-mentoring model. The potential of head-mounted displays in healthcare has yet been explored, with promising results in medical education and training [18]. However, no author has yet investigated the use of the MRH in a tele-mentoring setting. In our previous work, we explored the application of MRH in intraocular surgery, with encouraging results in applicability, education, safety and efficacy [10]. Here, we enhance and validate our previous results by using the MRH in a higher number of surgeries and testing intraoperative and postoperative parameters, to give us the possibility of developing the tele-mentoring model. Therefore, we defined and tested the tele-mentoring model, registering encouraging results. The necessity of developing an innovative model in surgical teaching relies on the ongoing conceptual shift due to upcoming novelties in ophthalmic surgery that call for change in education and training. Hence, the overall reported inequities in surgical teaching and surgical abilities of young ophthalmologists shed light on the need for innovative methods to improve surgical teaching. Previously, different studies have underscored inequities in surgical abilities among young ophthalmologists, calling for interventions in surgical training, monitoring and mentoring [19]. As a promising solution, the implementation with new MR and AR devices has been successfully tested in the pre-surgical training, and it is now part of the educational pattern in some realities [20]. Telemedicine has revolutionized the field of surgery, and tele-mentoring models have rapidly spread out in other specialties, embracing the importance of implementing VR and artificial intelligence in surgical teaching [21]. In this regard, telesurgery and tele-mentoring have been outlined as promising applications, allowing surgeons to assist or supervise others through mentoring from a different location [21]. Previously, 3D live-casting technique has been tested in teaching and mentoring settings, with the possibility of broadcasting microsurgeries live worldwide [9]. Likewise, Lucatto et al. have explored the role of tele-mentoring as supplementary tool for surgical training in scleral fixation surgery achieving a 95.5% success [22]. All this supporting research evidences the wide range of possibilities in the educational process related to the use of MRH. Recently, Schwartz and colleagues reported the use of a different Three-dimensional head-mounted digital visualization platform in vitreoretinal surgery, highlighting potential advantages despite the necessity of addressing limitations such as image quality issues. Nevertheless, to date, the aforementioned device is not suitable for the tele-mentoring model, due to the absence of a long-distance sharing platform [23]. As easily accessible, not geographically limited and continuously updated platforms, MR devices appear to be promising tool in surgical education [24]. By re-shaping the mentor-mentee communications, the tele-mentoring educational method boosts self-confidence, increases the sense of trust and responsibility still preserving the patient safety guaranteed by a supervised surgery. The mentor- mentee relation has overall been acknowledged as solid ground to grow and maximize the fellow surgical abilities. By playing a pivotal role in surgical training, mentorship should be programmed and established by analyzing and re-shaping it with time changes [25]. In fact, in contemporary times, this relation may be reinterpreted in a tele-mentoring reality, that does not require the mentor presence next to the mentee, leaving to more responsibility and confidence making the mentor-mentee relationship a game changer in fostering the fellow professional growth and success in the unique setting of the ophthalmic surgical environment [11]. 

The positive results in safety, efficacy and education registered in our analysis point out the suitability of the tele-mentoring model in the upcoming years as integrated part of the surgical training for Ophthalmology trainees and fellows. However, we acknowledge the necessity of further studies to validate our preliminary results in this innovative teaching method and to better elucidate its role in the context of a traditional surgical teaching.

In fact, long term, prospective studies should test the learning curve deriving from the application of the MRH in VR training. Nevertheless, our results in safety and efficacy are encouraging, despite the longer intraoperative times registered by the fellow during ROSO procedure that may be attributable to the necessity of transitioning to a new approach, and therefore may further be reduced by the learning curve. A recent review, has investigated the role of the mentor, which nowadays transcends the role of the supervisor, embodying an experienced and skilled advisor [11]. Surgical training is a resource-intensive process, necessitating significant time and investment [24]. Moreover, the acquisition of surgical skills often requires a high volume of cases under the supervision of trained surgeons. By uncoupling the mentor from the operating microscope, the resource allocation and planning process is improved, and a step forward is made in the fellow educational process. Contextually, the patient safety is preserved by performing the procedure under the mentor supervision. In fact, surgical efficacy and patient safety were pivotal in our model. While these two fields have already been explored in our previous publication, here we validated the technique with a larger sample, a longer follow-up and the analysis of intraoperative and postoperative parameters, and translated the tele-mentoring model proposal in reality [10]. Nevertheless, the method advantages are not only limited the fellows educational process. Hence, the possibility of transmitting at longer distances, and potentially be used as tool to implement surgical education worldwide. However, despite promising results obtained in this analysis we must acknowledge that the possibility of using this model as educational worldwide tool to support the surgeon abilities development in low income countries should be regarded taking into account technical limitations that may limit their applicability (ig connection instability, data security, communication redundancy and tolerance to delay). As a result, to test its full applicability in this setting, further studies should be conducted with strict methodological measures aligned with this purpose. A previous paper has advocated for sustainable and equitable development in global surgical care, reporting young ophthalmologists in low-income countries to lack in surgical training and resources [24]. In this regard, the authors evaluated the role of tele-mentoring in improving confidence and microsurgical skill among participants, enhancing its easy accessibility, continuous update, effectiveness and the possibility of distance surgical mentorship. In this light, by reporting safety and efficacy of the described method, our tele-mentoring method can potentially be considered as a valuable tool able to provide access to educational resources for surgical trainees all around the world, and in the development of educational training in low-resource settings. In this light, the possibility of mentoring young surgeons from far away opens to a wide range of educational programs, despite requiring the presence of experienced surgeons in situ to guarantee patient safety. However, we acknowledge the necessity of structurally adapting the model in a process and reality that could potentially take time and require finance. In fact, previous reports have outlined signal latency as significant issue affecting the outcome of tele-surgery, therefore underscoring the necessity of a reliable bandwidth network connection [26]. It is important to specify that our model is a feasibility analysis that poses the basis of an innovative tele-mentoring surgical model to implement and broaden the experience of surgical teaching, thus offering the fellow the possibility of training in different settings than the traditional one ( that is still fundamental in surgical teaching), enriched by the advantages yet exposed in visualization and surgery implementation. However, this has limitations. Firstly, the prerequisite of a strong connection that should be available in all involved settings. Secondly, the requirement of improving spaces and staff in the transition to the innovative surgical approach. Furthermore, we acknowledge that some limitations may be associated with the use of the MRH such as low battery life, limited memory size, and possible side effects (ig nausea and vomiting) however, none of these were present in our analysis. Additionally, the inability to quantify the time lag should be acknowledged as a limitation of this study. Finally, future model improvement should consider a straight communication between the MRH and the microscope, to allow the surgeon to directly see the operatory field in the viewer.

As advancements in technology and telecommunication provide us with infinite ways to re-imagine the relationship between people and healthcare, while time passes and changes, keeping posted with novelty is imperative in surgery and surgical education. As a result, despite changes in new realities and new approaches, education should continue moving on, keeping patient safety and surgical efficacy as pivotal. In this scenario, our tele-mentoring model is a valuable tool to shape the future ophthalmic surgeons.

## Supplementary Information

Below is the link to the electronic supplementary material.

Supplementary Material 1: Video 1. The video reports highlights of the tele-mentoring model based on the MRH. Once the Facetime call starts at the beginning of the surgery, the connection between surgeons is established, to allow the continuous update and discussion over the surgical steps, that are correctly being performed under the surgeon supervision over Facetime. The images shown refer to the remote surgeon smartphone interface (screen recording). The remote surgeon sees the MRH view (surgeon in the theatre) in the upper part of the screen where the all-in one platform is evident, showing the multimodal imaging of the surgical case. Thereafter, the remote surgeon shifts to the only surgeon’s view, to better appreciate the surgical steps.

## Data Availability

Due to privacy reasons, the datasets used and/or analyzed during the current study are available from the corresponding author on reasonable request.

## Abbreviations

MRH: Mixed reality headset
MR: Mixed reality
AR: Augmented reality
VR: Virtual reality
ROSO: Silicone oil removal
PPV: Pars Plana Vitrectomy
VH: Vitreous hemorrhage
ERM: Epiretinal membrane
FAF: Fundus autofluorescence
OCT: Optical coherence tomography
OR: Operatory room
